# De novo induction of tertiary lymphoid structures: an immunotherapeutic strategy in pancreatic cancer

**DOI:** 10.1038/s41392-025-02260-5

**Published:** 2025-05-26

**Authors:** Azaz Ahmed, Christoph Springfeld, Niels Halama

**Affiliations:** 1 Division of Tumor Immunology and Tumor Immunotherapy, German Cancer Research Center (DKFZ), Helmholtz-Institute for Translational Oncology (HI-TRON), Mainz, Germany; 2 Department of Medical Oncology, National Center for Tumor Diseases (NCT), University Hospital Heidelberg, Heidelberg, Germany; 3 Department of Hematology, Medical Oncology and Pneumology, University Medical Center of the Johannes-Gutenberg-University of Mainz, Mainz, Germany

**Keywords:** Gastrointestinal cancer, Immunotherapy

The recent publication by Amisaki et al.^1^ in *Nature* highlights a novel and druggable mechanism by which IL-33-activated innate lymphoid cells type 2 (ILC2s) induce the formation of tertiary lymphoid structures (TLSs) within the tumor microenvironment (TME) of pancreatic cancer. These findings open new avenues for therapeutic strategies aimed at enhancing TLS-mediated antitumor immunity and suggest lymphoneogenesis as a potential tool for immunotherapy.

Pancreatic ductal adenocarcinoma (PDAC) is among the most aggressive malignancies with a 5-year-survival rate of 8%.^2^ Despite advances in surgical techniques and chemotherapies, effective treatment options remain limited. The poor prognosis is largely attributed to the late diagnosis, highly invasive nature, and pronounced resistance to immunotherapy. The profoundly immunosuppressive TME restricts endogenous antitumor immune responses as well as the efficacy of immunotherapies such as immune checkpoint inhibition.

TLSs are ectopic lymphoid organs that form de novo in response to inflamed tissues including tumors. Their level of organization can vary widely, ranging from simple clusters of lymphoid cells - such as B and T cells alongside antigen-presenting cells - to fully developed lymphoid structures with organized zones of B and T cells, (follicular) dendritic cells and specialized blood vessels such as high endothelial venules.^3^ Notably, the presence of TLSs has been correlated with improved survival across multiple cancer types, including PDAC.^4,5^ However, inflammatory signals and cells responsible for TLS induction remain unidentified.

In their study, Amisaki et al. sought to address this question by first investigating expression patterns of genes that correlated with multiple known TLS signatures in PDAC. By this approach, they found that IL-33 expression correlated with all TLS signatures and with the expression of *LTB* (a gene encoding the TLS-promoting cytokine LTβ) in multiple PDAC cohorts. IL-33^+^ (immune) cells were primarily found within TLSs in tumors, and high densities of IL-33^+^ cells were associated with improved survival rates. After the identification of IL-33 as a potential key factor, a causal connection of IL-33 and TLS neogenesis was demonstrated utilizing a PDAC mouse model. Only wild-type PDAC mice, but not *Il33*^-/-^ mice showed tumor control and induction of TLSs upon activation of LTβ receptor, which is a typical signal for TLS neogenesis. By employing another PDAC mouse model and the administration of recombinant IL-33 (rIL-33), Amisaki and colleagues revealed that IL-33 acts as a trigger for TLS formation. rIL-33 induced intratumoral TLSs across various PDAC mouse models, promoting different maturations states (lymphoid aggregates, primary follicles). Another notable finding was a shift in the intratumoral B cell compartment (increased B cell frequency, B cell clonal expansion, somatic hypermutation) indicating potential germinal center reactions. These IL-33-dependent effects on inflammation were observed in PDAC and a colitis model.

To further elucidate mechanisms of TLS formation, the researchers aimed to identify the cells which mediate IL-33 driven lymphoneogenesis. Amisaki et al. elegantly used single-cell transcriptomics and identified an intratumoral population of ST2^+^ ILC2s co-expressing *Klrg1* and several lymphoid tissue-forming genes (*Ltb*, *LTa*, *LTα1/β2*), which are essential for TLS neogenesis. Moreover, rIL-33 specifically expanded KLRG1^+^ ILC2s, suggesting that these cells play a central role in IL-33-mediated TLS induction. This finding was further supported by innovative experiments using transgenic mice with selectively depleted ILC2s. Of note, intratumoral TLSs were significantly reduced in ILC2-depleted mice during rIL-33 treatment. Further, sophisticated loss- and gain-of-function experiments in multiple PDAC mouse models demonstrated that the LT signaling pathway in rIL-33-activated KLRG^+^ ILC2s drives TLS formation. These cells serve as inducer cells which need LTβR^+^ organizer cells as a binding target to coordinate lymphoneogenesis. CD11b^+^ myeloid cells were identified as such organizer cells expressing high levels of intratumoral LTβR with a TLS typical transcriptome profile (*Cxcl13*, *Ccl19*, *Vcam1*). Collectively, a fine-tuned cooperation of KLRG^+^ ILC2s and myeloid cells orchestrates IL-33-dependent TLS induction in PDAC.

Extending their findings, Amisaki et al. utilized congenic parabiotic mice with PDACs and demonstrated that administration of rIL-33 to donor parabionts triggered migration of KLRG1^+^ ILC2s into the bloodstream and tumors of recipient parabionts. After establishing the hematogenous migration of inducer KLRG1^+^ ILC2s to tumors, the researchers aimed to locate the origin of the cells. It was uncovered that PDACs modulate gut microbiota increasing gut *Il33* mRNA expression suggesting that a gut-blood-PDAC circuit is one potential route of migration of KLRG1^+^ ILC2s towards tumors. Another set of parabiosis experiments demonstrated that rIL-33 leads to a microbiota-mediated hematogenous migration of KLRG1^+^ ILC2s from pancreatic PDACs in donor mice to subcutaneous PDACs in recipient mice.

These mechanistic insights were obtained from preclinical models. Therefore, validating these findings in humans is crucial to enable clinical translation, especially with a focus on two aspects: the confirmation of the identified (innate) immune cell populations and their central role and the delineation of the microbiome (communities) in humans. Amisaki et al. were able to recapitulate that LT-expressing KLRG1^+^ ILC2s are detected in human PDACs and that activated ILC2 signatures correlate with TLS transcriptional signatures. This finding indicated that interventions in the IL-33-ILC2-TLS pathway could be an immunotherapeutic approach in human PDAC patients. A human version of rIL-33 (H-rIL-33) was engineered and tested in mouse models, demonstrating its ability to expand intratumoral KLRG1^+^ ILC2s and TLSs leading to tumor control. Activity in humans or patient-based models remains to be tested.

**Fig. 1:**
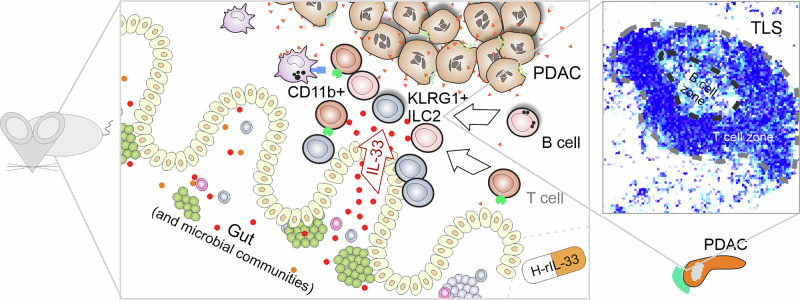
Overview with central proposed elements of tertiary lymphoid structure (TLS) lymphoneogenesis in mouse models. IL-33 produced in the gut of PDAC-bearing mice leads to migration of KLRG1^+^ ILC2 cells towards the tumor and interaction with CD11b^+^ myeloid helper cells. This in turn attracts B cells (and T cells) to form TLSs (as shown in the schematic pixelated TLS representation). The induction of TLSs was recapitulated in mice treated with the human drug candidate H-rIL-33

This pioneering study provided robust insights into the potential mechanisms of TLS formation and demonstrated how targeting this pathway could offer a therapeutic benefit for PDAC patients by enhancing anti-tumor immunity (Fig. 1). As part of this research, a potential human drug candidate, H-rIL-33, was developed and showed promising effects in mice. Moving forward, the key challenge will be determining whether the same TLS-inducing pathway exists in humans and whether clinical trials can validate the efficacy of an IL-33-based therapy in boosting anti-tumor immunity and improving patient outcomes in PDAC.
